# Supplementary material for: NSF-mediated disassembly of on- and off-pathway SNARE complexes and inhibition by complexin
Source: eLife. 2018 Jul 9;7:e36497. doi: 10.7554/eLife.36497 (PMC6130971; doi:10.7554/eLife.36497)
Supplement: Figure 2—source data 1. [file elife-36497-fig2-data1.pdf]

Figure 2—source data 1. Data summary table for the results shown in Figure 2D.

Conditions	Percent of molecules without transitions	Percent of molecules with transitions	Number of molecules analyzed	Number of fields of view
Alone (none)	27.2 ± 2.6	N.A.	1774	3
All (NSF/αSNAP/ATP/$MgCl_2$)	5.5 ± 1.3	15.8 ± 4.3	2892	4
No αSNAP (NSF/ATP/$MgCl_2$)	35.8 ± 1.4	N.A.	1572	3
No NSF (αSNAP/ATP/$MgCl_2$)	32.4 ± 5.3	N.A.	1714	3
No $MgCl_2$ (NSF/αSNAP/ATP)	31.1 ± 5.7	N.A.	1961	3
