# Supplementary material for: NSF-mediated disassembly of on- and off-pathway SNARE complexes and inhibition by complexin
Source: eLife. 2018 Jul 9;7:e36497. doi: 10.7554/eLife.36497 (PMC6130971; doi:10.7554/eLife.36497)
Supplement: Figure 3—source data 2. [file elife-36497-fig3-data2.pdf]

Figure 3—source data 2. Data summary table for the results shown in Figure 3D-E.

NaCl concentration (mM)	High FRET dwell time		Low FRET dwell time		Number of analyzed transitions
	Long-lived state population (%)	Short-lived state population (%)	Long-lived state population (%)	Short-lived state population (%)	
50	74.3 ± 3.1	25.7 ± 3.1	79.3 ± 2.7	20.7 ± 2.7	2481
100	76.3 ± 1.8	23.7 ± 1.8	77.3 ± 1.0	22.7 ± 1.0	3066
200	73.7 ± 5.0	26.3 ± 5.0	67.3 ± 2.2	32.7 ± 2.2	926
400	81.1 ± 4.0	18.9 ± 4.0	40.8 ± 1.8	59.2 ± 1.8	285
