# Supplementary material for: NSF-mediated disassembly of on- and off-pathway SNARE complexes and inhibition by complexin
Source: eLife. 2018 Jul 9;7:e36497. doi: 10.7554/eLife.36497 (PMC6130971; doi:10.7554/eLife.36497)
Supplement: Figure 3—source data 4. [file elife-36497-fig3-data4.pdf]

Figure 3—source data 4. Data summary table for the results shown in Figure 3I-J.

αSNAP concentrations (μM)	High FRET dwell time		Low FRET dwell time		Number of analyzed transitions
	Long-lived state population (%)	Short-lived state population (%)	Long-lived state population (%)	Short-lived state population (%)	
0.5	61.9 ± 3.4	38.1 ± 3.4	39.9 ± 11.4	60.1 ± 11.4	268
5.0	68.4 ± 3.5	31.6 ± 3.5	63.6 ± 6.2	36.4 ± 6.2	1266
10.0	76.3 ± 1.8	23.7 ± 1.8	77.3 ± 1.0	22.7 ± 1.0	3066
20.0	67.4 ± 0.2	32.6 ± 0.2	71.8 ± 5.3	28.2 ± 5.3	3490
