# Supplementary material for: NSF-mediated disassembly of on- and off-pathway SNARE complexes and inhibition by complexin
Source: eLife. 2018 Jul 9;7:e36497. doi: 10.7554/eLife.36497 (PMC6130971; doi:10.7554/eLife.36497)
Supplement: Figure 4—source data 2. [file elife-36497-fig4-data2.pdf]

Figure 4—source data 2. Data summary table for the results shown in Figure 4E-F.

αSNAP mutants	High FRET dwell time		Low FRET dwell time		Number of analyzed transitions
	Long-lived state population (%)	Short-lived state population (%)	Long-lived state population (%)	Short-lived state population (%)	
WT	76.3 ± 1.8	23.7 ± 1.8	77.3 ± 1.0	22.7 ± 1.0	3066
EEED	65.5 ± 7.7	34.5 ± 7.7	42.9 ± 8.5	57.1 ± 8.5	653
