# Supplementary material for: NSF-mediated disassembly of on- and off-pathway SNARE complexes and inhibition by complexin
Source: eLife. 2018 Jul 9;7:e36497. doi: 10.7554/eLife.36497 (PMC6130971; doi:10.7554/eLife.36497)
Supplement: Figure 5—source data 1. [file elife-36497-fig5-data1.pdf]

Figure 5—source data 1. Data summary table for the results shown in Figure 5D.

Construct	Syntaxin-1A residues	Percent of molecules without transitions	Percent of molecules with transitions	Number of molecules analyzed	Number of fields of view
L-SNARE-CC	181-262	5.5 ± 1.3	15.8 ± 4.3	2892	4
L-SNARE$_{full}$-CC	1-263	6.83 ± 0.6	13.8 ± 1.7	2648	3
