# Supplementary material for: NSF-mediated disassembly of on- and off-pathway SNARE complexes and inhibition by complexin
Source: eLife. 2018 Jul 9;7:e36497. doi: 10.7554/eLife.36497 (PMC6130971; doi:10.7554/eLife.36497)
Supplement: Figure 5—source data 2. [file elife-36497-fig5-data2.pdf]

Figure 5—source data 2. Data summary table for the results shown in Figure 5E-F.

Construct	High FRET dwell time		Low FRET dwell time		Number of analyzed transitions
	Long-lived state population (%)	Short-lived state population (%)	Long-lived state population (%)	Short-lived state population (%)	
L-SNARE-CC	76.3 ± 1.8	23.7 ± 1.8	77.3 ± 1.0	22.7 ± 1.0	3066
L-SNARE$_{full}$-CC	71.0 ± 1.3	29 ± 1.3	78.1 ± 3.2	21.9 ± 3.2	1661
