# Supplementary material for: NSF-mediated disassembly of on- and off-pathway SNARE complexes and inhibition by complexin
Source: eLife. 2018 Jul 9;7:e36497. doi: 10.7554/eLife.36497 (PMC6130971; doi:10.7554/eLife.36497)
Supplement: Figure 6—source data 2. [file elife-36497-fig6-data2.pdf]

Figure 6—source data 2. Data summary table for the results shown in Figure 6E-F.

Added protein	High FRET dwell time		Low FRET dwell time		Number of analyzed transitions
	Long-lived state population (%)	Short-lived state population (%)	Long-lived state population (%)	Short-lived state population (%)	
None	81.4 ± 1.7	18.6 ± 1.7	71.1 ± 1.9	28.9 ± 1.9	2804
1 µM Cpx WT	77.8 ± 11.2	22.2 ± 11.2	57.7 ± 13.7	42.3 ± 13.7	524
10 µM Cpx WT	75.6 ± 12.0	24.4 ± 12.0	29.2 ± 5.2	70.8 ± 5.2	1434
10 µM Cpx 4M	70.0 ± 5.6	30.0 ± 5.6	68.4 ± 3.8	31.6 ± 3.8	963
