# Supplementary material for: NSF-mediated disassembly of on- and off-pathway SNARE complexes and inhibition by complexin
Source: eLife. 2018 Jul 9;7:e36497. doi: 10.7554/eLife.36497 (PMC6130971; doi:10.7554/eLife.36497)
Supplement: Figure 7—source data 2. [file elife-36497-fig7-data2.pdf]

Figure 7—source data 2. Data summary table for the results shown in Figure 7H-I.

Construct	High FRET dwell time		Mid FRET dwell time		Low FRET dwell time		Number of analyzed transitions
	Long-lived state population (%)	Short-lived state population (%)	Long-lived state population (%)	Short-lived state population (%)	Long-lived state population (%)	Short-lived state population (%)	
L-SNARE$_{binary}$-CC1	83.6 ± 4.5	16.4 ± 4.5	57.8 ± 7.4	42.2 ± 7.4	71.9 ± 5.7	28.1 ± 5.7	2579
L-SNARE$_{binary}$-CC2	82.4 ± 3.8	17.6 ± 3.8	63.9 ± 8.5	36.1 ± 8.5	65.5 ± 1.3	34.5 ± 1.3	3319
