# Supplementary material for: NSF-mediated disassembly of on- and off-pathway SNARE complexes and inhibition by complexin
Source: eLife. 2018 Jul 9;7:e36497. doi: 10.7554/eLife.36497 (PMC6130971; doi:10.7554/eLife.36497)
Supplement: Figure 8—source data 2. [file elife-36497-fig8-data2.pdf]

Figure 8—source data 2. Data summary table for the results shown in Figure 8E-F.

Constructs	High FRET dwell time		Low FRET dwell time		Number of analyzed transitions
	Long-lived state population (%)	Short-lived state population (%)	Long-lived state population (%)	Short-lived state population (%)	
L-SNARE-CC	76.3 ± 1.8	23.7 ± 1.8	77.3 ± 1.0	22.7 ± 1.0	3066
L-SNARE-NN	81.6 ± 3.3	18.4 ± 3.3	77.3 ± 2.3	22.7 ± 2.3	975
L-SNARE$_{ternary}$-CC1	73.9 ± 2.0	26.1 ± 2.0	71.8 ± 2.4	28.2 ± 2.4	2851
L-SNARE$_{ternary}$-CC2	78.6 ± 5.1	21.4 ± 5.1	79.3 ± 3.0	20.7 ± 3.0	785
