# Supplementary material for: NSF-mediated disassembly of on- and off-pathway SNARE complexes and inhibition by complexin
Source: eLife. 2018 Jul 9;7:e36497. doi: 10.7554/eLife.36497 (PMC6130971; doi:10.7554/eLife.36497)
Supplement: Figure 9—source data 2. [file elife-36497-fig9-data2.pdf]

Figure 9—source data 2. Data summary table for the results shown in Figure 9F-G.

Construct	High FRET dwell time		Low FRET dwell time		Number of analyzed transitions
	Long-lived state population (%)	Short-lived state population (%)	Long-lived state population (%)	Short-lived state population (%)	
L-SNARE-CN	45.8 ± 1.7	54.2 ± 1.7	76.3 ± 7.0	23.7 ± 7.0	895
